# Pine Response to Sawfly Pheromones: Effects on Sawfly’s Oviposition and Larval Growth

**DOI:** 10.3390/insects15060458

**Published:** 2024-06-19

**Authors:** Asifur Rahman-Soad, Norbert Bittner, Monika Hilker

**Affiliations:** 1 Applied Zoology/Animal Ecology, Institute of Biology, Freie Universität Berlin, 12163 Berlin, Germany; m.rahman.soad@fu-berlin.de (A.R.-S.); norbit@zedat.fu-berlin.de (N.B.); 2 Institute of Translational Genomics, Helmholtz Zentrum München Deutsches Forschungszentrum für Gesundheit und Umwelt, 85764 Neuherberg, Germany

**Keywords:** pine, sawfly, herbivore, insect egg, sex pheromone, oviposition, behavior, olfaction, co-evolution

## Abstract

**Simple Summary:**

Mass outbreaks of the pine sawfly *Diprion pini* can cause severe damage to pine forests. The larvae of this herbivorous insect feed selectively on the needles of pine trees, notably *Pinus sylvestris.* During mass outbreak periods, the females release large amounts of sex pheromones. A prior study revealed that the survival rate of sawfly eggs laid on pheromone-exposed pine needles was lower than that of eggs on unexposed pine. In our study, we found that *D. pini* females avoided oviposition on pheromone-exposed pine, possibly as a counter-adaptation to the enhanced defenses of previously pheromone-exposed trees against sawfly eggs. The females only discriminated between pheromone-exposed and unexposed trees when they had the chance to touch the needles, but not when exposed to the odor of these types of trees. However, the performance of larvae did not significantly differ on pheromone-exposed and unexposed trees. These results underscore the complexity of the chemical ecology of sawfly–pine interactions and highlight the nuanced roles that pheromones play in shaping the relationships between herbivores and their host plants.

**Abstract:**

Insect pheromones have been intensively studied with respect to their role in insect communication. However, scarce knowledge is available on the impact of pheromones on plant responses, and how these in turn affect herbivorous insects. A previous study showed that exposure of pine (*Pinus sylvestris*) to the sex pheromones of the pine sawfly *Diprion pini* results in enhanced defenses against the eggs of this sawfly; the egg survival rate on pheromone-exposed pine needles was lower than that on unexposed pine. The long-lasting common evolutionary history of *D. pini* and *P. sylvestris* suggests that *D. pini* has developed counter-adaptations to these pine responses. Here, we investigated by behavioral assays how *D. pini* copes with the defenses of pheromone-exposed pine. The sawfly females did not discriminate between the odor of pheromone-exposed and unexposed pine. However, when they had the chance to contact the trees, more unexposed than pheromone-exposed trees received eggs. The exposure of pine to the pheromones did not affect the performance of larvae and their pupation success. Our findings indicate that the effects that responses of pine to *D. pini* sex pheromones exert on the sawfly eggs and sawfly oviposition behavior do not extend to effects on the larvae.

## 1. Introduction

Chemical communication via pheromones plays an important role in the insect world. Insect pheromones convey complex information between conspecific individuals about, e.g., the presence of conspecifics, mating partners, territorial boundaries, paths to resources, or danger [1,2,3,4]. Insect pheromones, which are perceived by olfactory receptors, are volatile compounds of various chemical structures [5,6]. While a plethora of studies have addressed the ecology, physiology, and molecular basis of insect communication via volatile pheromones [2,7,8,9], scarce knowledge is available on how these chemicals affect plant responses, which in turn may shape the insect’s behavior, development, or survival.

Recent advancements have shed light on how the exposure of a plant to volatile insect pheromones affects that plant’s defenses against herbivorous insects. Helms et al. [10,11] were the first to show that plant perception of an insect pheromone results in improved plant defense responses to subsequent insect infestation. In general, cues that “warn” a plant of impending stress and result in subsequent, improved stress responses are referred to as “priming” stimuli [12,13]. Defense priming is a process which prepares the plant to respond more rapidly or more intensively to future attack [13,14,15]. In addition to insect pheromones, several other cues are known to prime plant anti-herbivore defenses, among them being herbivory-induced and oviposition-induced plant volatiles (HIPVs and OIPVs), insect eggs, and vibrations caused by leaf-feeding larvae [16,17,18,19]. The research by Helms et al. [10] on goldenrod plants (*Solidago altissima*) revealed that exposure of the plants to volatiles released from male goldenrod gall flies, *Eurosta solidaginis*, elicited enhanced the anti-herbivore defense responses of the plant, resulting in reduced feeding damage by a leaf beetle specialized upon goldenrod plants. Pheromone-exposed, feeding-damaged goldenrod plants showed higher levels of jasmonic acid than unexposed, feeding-damaged plants. The volatile compound released by male *E. solidaginis* that evokes the enhanced feeding-induced defense in goldenrod plants was identified as *E*,*S*-conophthorin, a spiroacetal [11]. Interestingly, goldenrod gallfly females avoided egg deposition onto previously pheromone-exposed plants.

Similarly, exposure of Scots pine (*Pinus sylvestris*) to the sex pheromone components of the herbivorous pine sawfly *Diprion pini* were shown to affect the tree’s defense responses [20]. Pine trees exposed to these pheromones showed improved defense against eggs of this sawfly. Fewer eggs survived on trees exposed to the pheromonal components (2*S*,3*R*,7*R*)-3,7-dimethyl-2-tridecanyl acetate and propionate. These compounds can attract *D. pini* males over great distances [21,22], indicating that the pheromone can be widely transferred in a pine forest. Sawfly egg deposition induces accumulation of H_2_O_2_ in pine needles. The pheromone-exposed trees with sawfly eggs accumulated more H_2_O_2_ than unexposed trees with eggs, suggesting that ROS (reactive oxygen species) signaling is involved in this pheromone-mediated, improved plant defense against insect eggs [20].

Here, we addressed the question of whether *D. pini*, highly specialized on pine, evolved an oviposition behavior that allows it to cope with the host plant’s primability of defenses against the eggs. A strategy to enhance the survival rate of eggs would be avoidance of areas with high pheromone concentrations. However, a previous study showed that *D. pini* females do not physiologically respond to their own pheromones [20]. Thus, they cannot use them as an abundance sensor, as some lepidopteran species can do [23]. We tested whether *D. pini* females avoid oviposition on pheromone-exposed pine. Such an avoidance behavior might be due to deterring pheromone-induced pine volatiles or contact cues. Furthermore, we investigated whether the detrimental effect that pheromone-exposed pine exerts on the egg survival rate extends to detrimental effects on larvae that hatch from the surviving eggs.

In detail, we addressed the following questions:

Can females of *D. pini* discriminate between odor released from pheromone-exposed trees and odor from untreated trees?

Do females of *D. pini* oviposit as readily on pheromone-exposed trees as on untreated trees?

Does exposure of pine to the pheromones affect larval performance and pupation rate?

Answers to these questions may shed light on the pine–pine sawfly interactions, which developed over a long evolutionary time [24] and resulted in adaptations and counter-adaptations of both players, thus forming robust host plant–herbivore interactions.

## 2. Materials and Methods

### 2.1. Insect and Plant

*Diprion pini* were reared in a climate chamber under conditions of 20 °C, 18 h:6 h light:dark, and 70% relative humidity. Scots pine twigs were collected from the Grunewald Forest in the south-west of Berlin, placed in vases filled with tap water, and enclosed in a transparent 14.9 L Plexiglas^®^ cylinder (height: 50 cm, diameter: 19.5 cm), which was closed with a gauze lid on top. Adult *D. pini* were introduced into the setup for mating and egg deposition. Usually, 12 to 14 days after egg deposition, larvae began to hatch and started feeding on the needles. During larval development, larvae were provided with Scots pine twigs ad libitum. The larvae show five (male) to six (female) instars until pupation [25]. Upon the onset of pupation, the cocoons were collected and stored in a refrigerator at 4 °C in the dark.

For the experiments, three-year-old *P. sylvestris* trees (40 to 60 cm in height with a stem diameter ranging between 2 and 4 mm) were acquired from a local tree nursery (Baumschule Stackelitz GmbH & Co. KG; 06,868 Coswig/OT Stackelitz; Germany). These small trees were grown individually in pots filled with Classic T potting soil (Einheitserde^®^, a mixture of peat and clay; N = 340 mg·L^−1^, P_2_O_5_ = 260 mg·L^−1^, K_2_O = 330 mg·L^−1^). The trees were initially kept in a greenhouse under long-day conditions (20 °C, 18 h:6 h light:dark) until the start of the experiments. One week prior to the start of the experiments, the trees were transferred to a climate chamber for acclimation to the abiotic experimental conditions (20 °C, 18 h:6 h light:dark, 70% relative humidity). Each potted tree was enclosed in a transparent 14.1 L Plexiglas^®^ cylinder (height: 80 cm, diameter: 15 cm) with an air inlet at the bottom and an air outlet on top. Each cylinder was ventilated by charcoal-filtered air at a rate of 250 mL·min^−1^ through an inlet at the bottom; the air flowrate on top was the same, thus ensuring that only charcoal-filtered air was inside the cylinder. To prevent volatile organic compounds (VOCs) released from the soil or the roots from affecting aboveground pine responses, the pots were wrapped with polyethylene terephthalate (PET) bags covering the pots and soil.

Pine trees were exposed to the synthetic male-attracting sex pheromones of *D. pini* females, specifically (2*S*,3*R*,7*R*)-3,7-dimethyl-2-tridecanyl acetate and propionate, for treatments (supplied by the laboratory of Olle Anderbrant, Lund University, Sweden). The pheromone esters were dissolved in hexane, each at a concentration of 50 ng·μL^−1^.

For pine treatments with pheromones, 100 μL pheromone solution dissolved in hexane (50 μL acetate + 50 μL propionate) was applied to a cotton wool pad (5.6 cm diameter and 0.4 cm thickness) as a dispenser. Control trees were supplied with a solvent control (100 μL hexane on a cotton wool pad). To allow solvent evaporation, the cotton pads were kept under a fume hood for 30 min before exposing them to the trees. After this time, the highly volatile hexane was expected to have evaporated, and a pad with pheromones was placed into the aforementioned 14.1 L Plexiglas^®^ cylinder with a pine tree for 24 h. Additionally, for a second control (blank), an untreated cotton pad without any solvent was added to a cylinder with a pine tree. After a treatment period of 24 h, the pads were removed from the cylinders. The chosen amount of pheromones estimates an equivalent of 270 to 450 females per tree, which comes close to the actual numbers in a mass outbreak [20].

For the larval performance assays (compare Section 2.4), in addition to pheromone-treated, hexane-treated, and untreated trees, we also used trees that were exposed to the pheromone components and afterwards subjected to egg deposition by two *D. pini* females for a duration of three days; the females had the chance to mate with two *D. pini* males during this time. Thus, we could study larval performance on pheromone-treated and subsequently egg-laden pine. For a control, hexane-treated trees as well as “blank” trees were exposed to *D. pini* egg deposition and subsequently to the larval performance assay.

### 2.2. Olfactory Choice Assay

To test how *D. pini* females responded to the odor of pheromone-exposed trees, we conducted a choice experiment as described by Hilker & Weitzel [26]. Female *D. pini* were allowed to crawl upwards along a Y-shaped glass rod (length of base stem: 35 cm, length of diverging Y-legs: 20 cm; diameter of the stem and legs: 1 cm; distance and angle between Y-legs on top: 60 cm; 90°). The setup was illuminated with a light positioned 80 cm above the base of the Y-glass rod (Philips TL-E 29-530 bulb, 22 Watt), which encouraged the females to walk upwards readily. The bioassay was carried out at 20 °C room temperature.

An untreated control tree was placed at the end of one of the Y-legs and a pheromone-exposed tree at the end of the other Y-leg. While crawling upwards along the glass rod, females could not touch the plants. When a female reached the end of one of the legs, the choice was recorded. The maximum observation period per female was 5 min. Females that did not choose one of the Y-legs within this observation period were considered to have made no choice.

We tested plants one day, two days, and five days after treatment. For each time, three plant pairs were tested, with 11 to 13 females per plant pair. After having tested four to five females, the rod was turned by 180°. For each plant pair, a new clean glass rod was used.

### 2.3. Oviposition Assay

To evaluate the oviposition behavior of *D. pini* in response to pine trees treated with pheromones, as well as those treated with hexane or left untreated, a no-choice experiment was performed. In the end of the 24 h-treatment period of a pine tree (compare Section 2.1), two *D. pini* females and two males were introduced into a Plexiglas^®^ cylinder (Nordic Panel GmbH, Stade, Germany) containing a tree. Females and males were used that had emerged two to three days before from the cocoons and had no chance to mate prior to the assay. These insects were observed over a period of five days to assess their egg-laying activity. The abiotic conditions were 20 °C, 70% relative humidity, and 18 h:6 h light:dark. The experiment was repeated five times, with each round including an equal number of trees per tested group (*n* = 22 trees per group in total). After the five-day period, we recorded the number of trees with and without eggs, as well as the number of eggs on egg-laden trees.

### 2.4. Larval Performance Study

To investigate the effect of sawfly sex pheromones on pine defenses against larvae, we allowed larvae to feed on trees exposed to the pheromones, to the solvent hexane, or left untreated. Five larvae (two to three days old) were taken from the rearing setup and placed on each tree. Their weight was recorded after 3, 9, and 15 days of feeding. Post weighing them after a 15-day feeding period, larvae were transferred to new untreated trees to monitor their development until pupation. A transfer to new trees was necessary because the larvae had eaten up almost all needles from the trees onto which they initially had been placed. Transfer of larvae to new, untreated trees allowed the determination of how a 15-day feeding period on pheromone-trees affects pupation success.

A second larval performance study was conducted to explore if pine exposure to sawfly pheromones enhances egg-mediated anti-herbivore defenses. Therefore, *D. pini* larvae were allowed to feed on trees previously exposed to pheromones (or to hexane or left untreated) and subsequently treated with *D. pini* egg deposition (compare Section 2.1). Thirteen days post egg deposition, five *D. pini* larvae (two to three days old) were taken from the rearing and introduced to these trees. This timing aligns with the natural egg incubation period under the given abiotic conditions (20 °C, 70% relative humidity, and 18 h:6 h light:dark); in nature, hatching larvae start feeding at their natal site. To maintain a controlled number of five larvae feeding on the trees, we killed the eggs on the trees by piercing them with sterile needles shortly before the larvae could hatch.

Both larval performance studies were conducted in multiple repeats. Each repetition included trees from all different treatments, with five larvae per tree. Larvae were individually weighed, serving as biological replicates. Variability in biological replicates was due to larval mortality and recovery issues throughout the experiments.

### 2.5. Statistics

Graphing and data analysis were conducted using GraphPad Prism (V 10.2.1) (GraphPad Software, Boston, MA, USA). Initially, the data underwent a normality test using the Shapiro–Wilk method, followed by appropriate statistical tests.

The non-normally distributed data of the olfactory dual choice assay were evaluated by the Wilcoxon matched-pairs signed rank test. Since the data of the oviposition assays and the larval performance study were also non-normally distributed, we compared the data obtained with pheromone-exposed, hexane-exposed, and untreated pine with Kruskal–Wallis tests. In cases of significant treatment effects, a Kruskal–Wallis test was followed by Dunn’s tests for dual comparisons. Pairwise comparisons of the weight of larvae on egg-free and egg-laden pine trees (previously treated, or hexane-exposed, or pheromone-exposed) and of the larval pupation rate on these types of trees were conducted by Mann–Whitney *U* tests. *p*-values exceeding 0.05 were deemed not significant (ns), whereas *p*-values below 0.05 were classified as significant.

## 3. Results

### 3.1. Females of D. pini Do Not Discriminate between Odor from Pheromone-Exposed and Unexposed Pine

**Figure 1:**
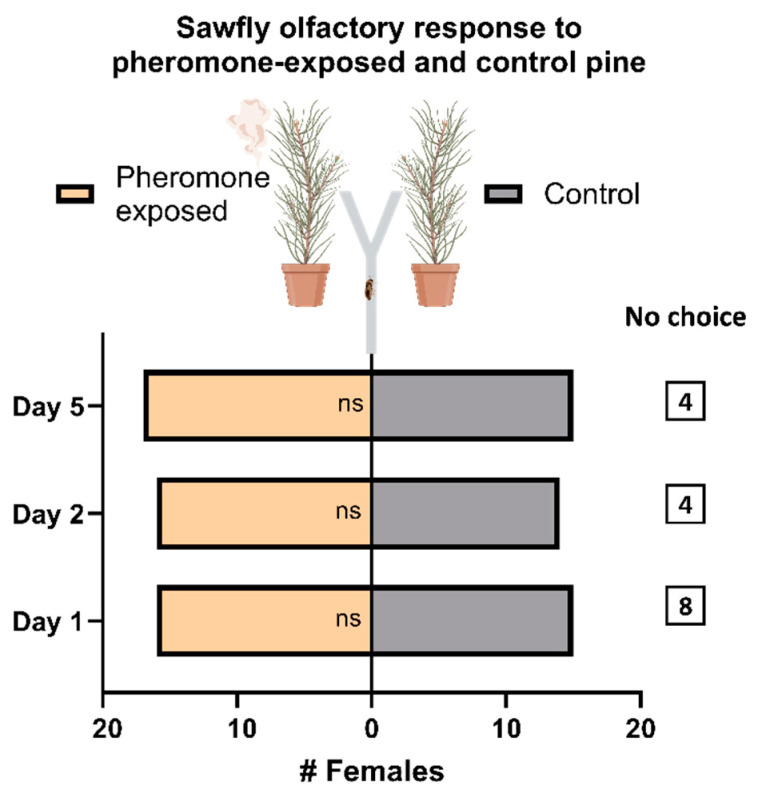
Responses of *Diprion pini* females to odor of pheromone-exposed and unexposed (control) *Pinus sylvestris* trees. Bioassay setup: Y-shaped glass rod: the end of one arm was equipped with a pheromone-exposed tree and the other arm with an untreated control tree. Female insects ascended the glass rod and selected one of the two Y-arms. The choice was evaluated at three time intervals: one, two, and five days after a 24 h-long pheromone exposure. Test period: five minutes. “No choice”: number of females that did not select either arm within the test period. Each test day included three pairs of plants, each with *n* = 11–13 *D. pini* females, resulting in a total of *n* = 34–39 females for each test day. Wilcoxon matched-pairs signed rank test; no significant (*p* > 0.05) differences between the number of females responding to the odor of the control tree and the pheromone-exposed tree (indicated by “ns”).

The olfactory choice assay revealed that the number of *D. pini* females that moved towards the odor released from pheromone-exposed *P. sylvestris* trees did not differ from the number of females that orientated towards the odor from unexposed trees. Regardless of the time past pheromone exposure (one, two, or five days), the results show no specific preference of the females for either the pheromone-exposed or control trees. A few females did not choose between the two odor sources within the observation period; they neither walked up the olfactometer Y-leg equipped with the pheromone-exposed tree nor the Y-leg with the control tree. They account for only 12–25% of all tested females when considering the three time intervals past pheromone exposure (Figure 1).

### 3.2. Ovipositing D. pini Females Prefer Trees without Prior Pheromone Exposure, but If Chosen, the Number of Eggs on Pheromone-Exposed Trees Does Not Differ from That on Unexposed Trees

**Figure 2:**
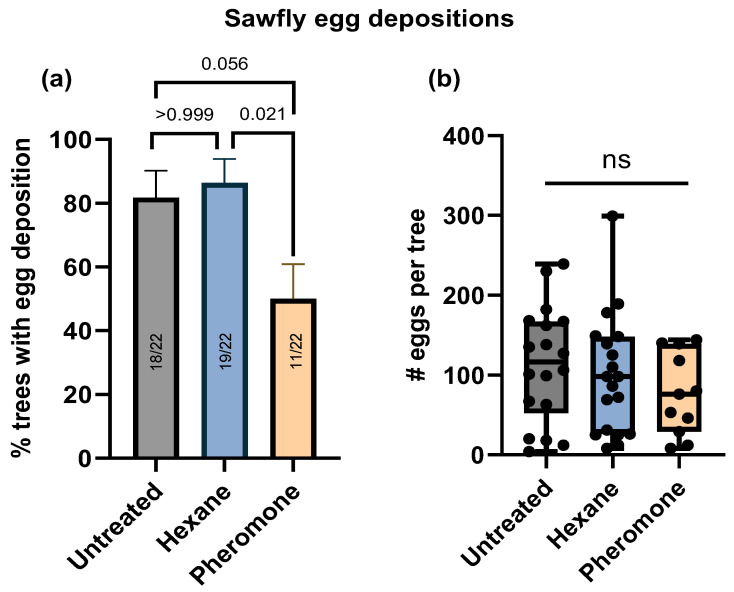
*Diprion pini* egg depositions. No-choice assay: an untreated, a hexane-exposed, or a pheromone-exposed *Pinus sylvestris* tree was offered separately to each two females and two males for five days. In total, *n* = 22 trees of each type were offered. (**a**) Mean percentage (±SE) of trees that received *D. pini* egg depositions and (**b**) number of eggs per tree that received egg deposition presented by box plots with medians, first and third quartiles, and full range of scores. (**a**,**b**) Kruskal–Wallis test followed by Dunn’s multiple comparisons; in (**a**), exact *p*-values are given; in (**b**), ns not significant (*p* ≤ 0.05).

When counting the number of trees that received *D. pini* eggs, the type of pine treatment significantly affected which tree was chosen for egg deposition (Figure 2a). Significant treatment effects (*p* = 0.0137) were detected by a Kruskal–Wallis test. Notably, a post hoc (Dunn’s) test revealed no significant difference between the number of untreated and hexane-treated trees with eggs (*p* > 0.9999), indicating that the solvent used to dissolve the pheromone had no additional effect. However, *D. pini* females were by trend less likely to oviposit on pheromone-exposed trees than on untreated trees (*p* = 0.0561). Furthermore, significantly fewer pheromone-exposed trees than hexane-treated trees received egg depositions (*p* = 0.0216). These data indicate that *D. pini* females preferably lay their eggs on trees that have not been exposed to pheromones.

When counting the number of eggs on trees that had been chosen by *D. pini* for egg deposition, no significant treatment effects were detected (*p* = 0.4705, Kruskal–Wallis test) (Figure 2b). These results suggest that while exposure of pine trees affects the initial selection of oviposition sites, the pheromone exposure does not significantly impact the overall number of eggs laid per tree (Figure 2b).

### 3.3. Performance of D. pini Larvae Is Not Affected by Exposure of Pine Trees to the Sawfly’s Pheromones, but by Pine Responses to Sawfly Egg Deposition

**Figure 3:**
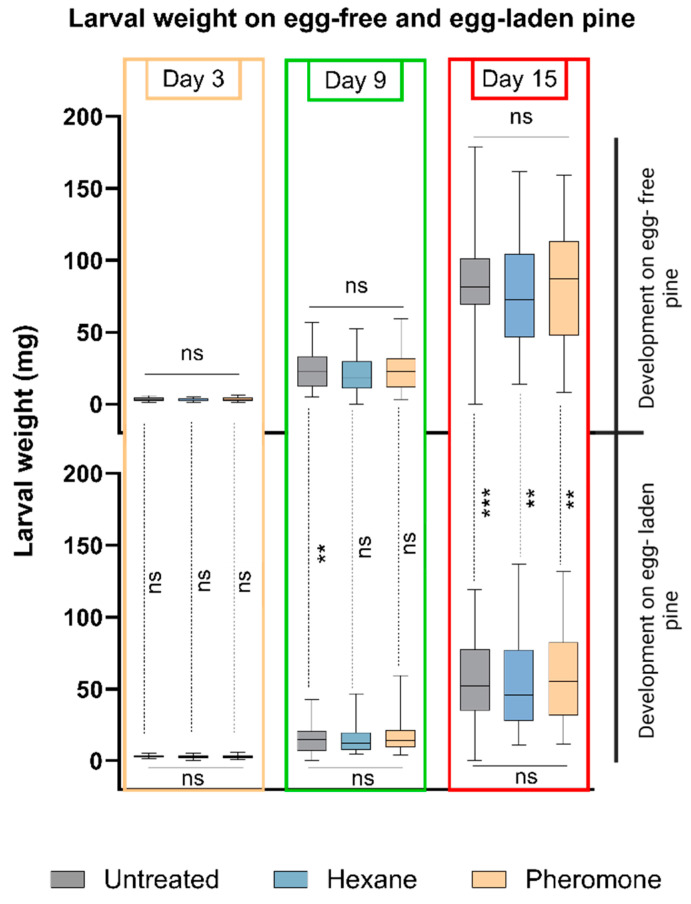
Weight of *Diprion pini* larvae on differently treated *Pinus sylvestris* trees. Types of trees offered without eggs (top) and with eggs (bottom): untreated, hexane-treated, and pheromone-exposed. Each treatment consisted of 7–10 trees and 5 larvae per tree. Young larvae were placed on a tree, and their weight was recorded after 3, 9, and 15 days of feeding. Bars show medians, first and third quartiles, and full range of larval weights. Within-day comparisons: Kruskal–Wallis test; ns not significant (*p* > 0.05). For comparison of larval weights on egg-free (top) and egg-laden trees (bottom): Mann–Whitney *U* test; significances indicated along vertical, dotted lines: ns not significant (*p* > 0.05), ** (*p* < 0.01), *** (*p* < 0.001).

Larvae of *D. pini* performed equally well on pheromone-exposed, hexane-exposed, and untreated trees. Their weight gain on these types of trees did not significantly differ. No effects of the pheromone or hexane exposure were detected, regardless of whether the trees had received *D. pini* eggs or not after the pre-treatment with pheromones or hexane (Figure 3).

However, when comparing the weight of larvae on trees with and without *D. pini* egg deposition, significant differences were detected. After a 15-day feeding period, larvae had gained more weight on egg-free pine trees than on previously egg-laden pine, regardless of the type of tree pre-treatment. When comparing the weight of larvae on previously untreated egg-free and egg-laden pine trees, a significant difference was detectable after a nine-day and fifteen-day feeding period (Figure 3). These findings suggests that pine responses to *D. pini* egg deposition enhance the tree’s defense against *D. pini* larvae, consistent with the observations by Beyaert et al. [27]. Our study here indicates that this egg-mediated defense against *D. pini* is independent of prior exposure to sawfly sex pheromones.

**Figure 4:**
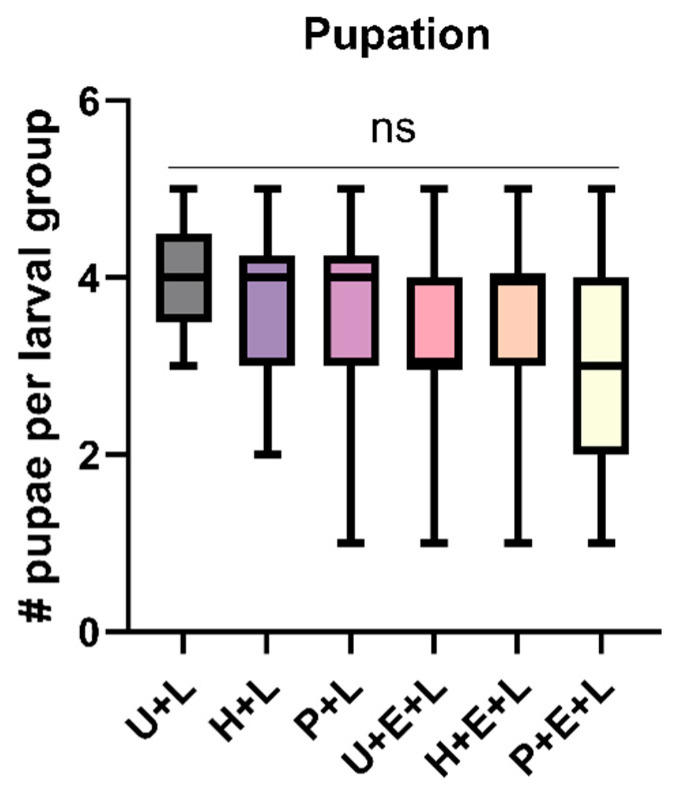
Number of pupae that resulted from a group of five *Diprion pini* larvae per tree. *Pinus sylvestris* trees with larvae were kept either untreated (U + L), hexane-exposed (H + L), or pheromone-exposed (P + L) and had no prior egg deposition; or the trees had received sawfly egg deposition prior to larval feeding and were left otherwise untreated (U + E + L), hexane-exposed (H + E + L), or pheromone-exposed (P + E + L) trees; *n* = 7–10 trees/treatment; 5 larvae/tree. Box plots with medians, first and third quartiles, and full range of scores are shown for each group. Kruskal–Wallis test; ns not significant (*p* > 0.05).

Furthermore, when extending observations to track larval development up to pupation, we found that the number of larvae that successfully pupated did not significantly differ between larvae on pheromone-exposed, hexane-exposed, and untreated trees, regardless of whether the trees had received eggs or not after the exposure to pheromones or hexane (Figure 4, Supplemental Table S1). Kruskal–Wallis tests revealed no significant treatment effects, neither within the egg-free group (*p* = 0.8986) nor within the egg-laden group (*p* = 0.4416). When comparing the numbers of successfully pupated larvae across all the tested conditions, no significant differences in pupation success were detected by a Kruskal–Wallis test (*p* = 0.287).

## 4. Discussion

Based on a previous study, which demonstrated that exposure of *P. sylvestris* to *D. pini* sex pheromones results in reduced egg survival rates, we investigated here whether *D. pini* females show a counter-adaptation to this egg-harming pine response to the pheromones. The results presented here provide evidence that sawfly females prefer egg deposition on pine that has not been exposed to these pheromones. We further addressed the question of whether the egg-harming response of pine to sawfly pheromones also affects larval performance. However, larval performance on pheromone-exposed pine did not significantly differ from the performance of larvae on trees that had not been exposed to the pheromones.

### 4.1. No Olfactory Discrimination by D. pini between Pheromone-Exposed and Unexposed Pine

The finding that the sawfly females did not discriminate between the odors of pheromone-exposed and untreated trees (Figure 1) might be due (i) to a lack of differences between the odor of untreated and pheromone-exposed pine, (ii) to the inability of *D. pini* females to discriminate between these odors, or (iii) to the type of olfactometer bioassay setup.

Ad (i): When considering the chemical nature of the *D. pini* pheromonal ester components, it is tempting to suggest that exposure of pine to them induces the release of pine volatiles. Plant responses to other carboxylic acid esters such as methyl salicylate, methyl jasmonate, or (*Z*)-3-hexenyl acetate have been shown to induce or prime anti-herbivore plant defenses [28,29,30,31], including those which rely on enhanced emission of plant VOCs, e.g., [28]. Whether exposure of pine to *D. pini* pheromones results in enhanced emission of terpenoid VOCs needs to be investigated by future chemical analyses comparing the volatile patterns of pheromone-exposed and untreated pine.

Ad (ii): Previous studies revealed that *D. pini* females are able to perceive pine volatiles (as might be expected from host plant volatiles) and to respond to them behaviorally, probably in a pine terpenoid-concentration dependent manner. High concentrations of pine terpenoid volatiles were shown to repel *D. pini* females [26]. However, this perception ability does not necessarily include the ability to detect possible differences in the odor of pheromone-exposed and untreated pine. Future studies need to elucidate the sawfly’s olfactory abilities to discriminate between different pine volatile blends.

Ad (iii): In our olfactometer bioassay, we tested whether *D. pini* females preferred the odor released by untreated or by pheromone-exposed pine from a distance of about 20 cm. We cannot exclude that possible preferences for one of these odor sources become evident only at a shorter range. For example, in *Drosophila sechellia*, different olfactory receptors are relevant in long- and short-range attraction to an odor source [32].

### 4.2. Oviposition Preference of Unexposed Pine over Pheromone-Exposed Pine upon Contact

While *D. pini* females did not olfactorily discriminate between pheromone-exposed pine and unexposed pine, they discriminated between these types of trees when ovipositing. For the oviposition bioassay, the females were enclosed with either a pheromone-exposed tree or an unexposed tree, without the option to move to a different tree during this experiment. While the females enclosed with an untreated tree readily laid their eggs, the females enclosed with a pheromone-treated tree obviously were reluctant to lay their eggs, resulting in a lower number of pheromone-exposed trees than unexposed trees with eggs (Figure 2a). However, when the females decided to lay eggs on pheromone-exposed pine, the number of eggs laid on these trees did not differ from the number on unexposed trees (Figure 2b). This latter result might have been due to a high oviposition pressure of those females laying their eggs on the pheromone-exposed trees; in the conducted no-choice assay, they had no alternatives. For some plant–insect interactions, it is known that eggs that have been laid onto leaves induce a change in plant quality, which affects the plant’s attractiveness for subsequent oviposition [33]. It is unknown so far how the changes that *D. pini* egg deposition induces in pine [20,25,34] affect the attractiveness of pine for subsequent sawfly egg deposition. Conifer sawflies are known to engage in a meticulous tactile examination of pine needles, involving walking up and down the needles and palpating them with their antennae, before egg laying [35]. Some species proceed to cut a ‘test-slit’ to further evaluate the needle quality [36,37]. Overall, our results suggest that the pheromone exposure changed tactile or close-range chemical cues in the final decision-making process for egg laying. While herbivorous insects often rely on plant volatiles when searching for host plants over long distances, contact cues may provide information about the suitability of the host [38,39,40]. Future studies should analyze whether pheromone-exposed pine needles exhibit changes in their cuticular chemical profile or patterns of volatiles relevant for close-range orientation of ovipositing *D. pini*.

Preference of unexposed over pheromone-exposed pine trees for egg deposition may be considered a counter-adaptation when taking into account that the egg survival rate on pheromone-exposed trees is significantly lower than on unexposed ones [20]. Exposure of goldenrod plants to the sex pheromone of male gallflies also reduced the number of gallfly ovipositions onto the pheromone-exposed plants when compared to unexposed plants [10]. However, whether exposure of goldenrod plants to gallfly pheromone results in reduced survival rates of gallfly eggs or reduced performance of gallfly larvae is unknown so far.

### 4.3. No Detrimental Effects of Pheromone-Exposed Pine on Larval Performance

While exposure of pine to *D. pini* sex pheromones affects sawfly oviposition behavior (Figure 2) and egg survival rate [20], it does not impact larval performance (Figure 3) and pupation success (Figure 4).

Numerous studies have investigated the relationship between insect oviposition preferences and offspring performance [41,42]. According to the preference-performance hypothesis (PPH) and “mother knows best” theory [43,44], females of herbivorous insects are expected to lay their eggs preferentially onto plants where the offspring will develop and survive the best. Most of these studies focus on the relationship between oviposition preferences and larval performance [45,46,47,48]. However, in addition to the larval development and survival, the egg survival rate also needs consideration, especially when considering the multitude of defensive plant responses to insect egg deposition [18].

Therefore, we suggest that oviposition preferences might be much more tightly associated with egg survival rates than larval survival rates. Indeed, several studies did not find support for a tight link between oviposition preference and larval performance [41,47,48]. In specialized insects, this might be due to a wide range of factors, among them being, e.g., the availability of optimal host plants, the risk of predation, parasitization, or pathogen infection of the larvae, and a mismatch of the suitability of the host plants for the egg laying female and for the larvae [49]. However, these factors may also affect the relationship between oviposition preference and egg survival rates. Under laboratory conditions, exposure of pine to *D. pini* pheromones was shown to lead to reduced egg survival [20]. If this effect of pheromone exposure also applies under natural conditions, we expect a close link between the sawfly’s oviposition preference and egg survival rate, even in forests.

Exposure of pine to *D. pini* pheromones is known to upregulate the expression of *PsRboh* (a pine respiratory burst oxidase homolog), indicating ROS signaling that may initiate further defense responses [50,51]. So far, we do not know whether pine changes its secondary metabolite profile or modifies its pattern of nutritious compounds in response to the exposure to *D. pini* pheromones. However, if so, *D. pini* larvae seem to be well adapted to such changes, since neither their weight nor their pupation success were affected when feeding upon pine trees that had been exposed to the pheromones (Figure 3 and Figure 4).

### 4.4. No Amplifier Effect of Pheromone Exposure on Egg-Mediated Improved Anti-Herbivore Defense

While exposure of pine to *D. pini* pheromones did not affect larval performance, our study showed that larvae gain less weight when feeding upon previously egg-laden pine (Figure 3). These results, obtained with sawfly larvae feeding upon juvenile pine trees, corroborate previous findings which also showed that *D. pini* performs worse when feeding upon previously egg-laden branches of mature (at least 15-year-old) pine trees [27]. Hence, our findings suggest that pine can take *D. pini* egg deposition as a highly reliable indication of impending larval herbivory, rendering any amplification of the egg-mediated improvement of pine defense against larvae via a further response to the pheromones redundant.

## 5. Conclusions and Perspectives

Our study provided further insights into how the sawfly *D. pini* can adjust its behavior based on the responses of its host plant to cues preceding and indicating impending infestation. We showed here that *D. pini* females are reluctant to lay their eggs on pheromone-exposed pine, thereby escaping from the enhancer effect of pheromone exposure on pine defense against sawfly eggs. In contrast, larval performance was not affected by exposure of pine to sawfly pheromones. Future studies need to elucidate whether pine does not change its nutritive quality in response to the pheromones or whether larvae are well adapted to such potential changes. Furthermore, a comparison of the odor profiles of pheromone-exposed and unexposed pine needs to elucidate whether pine changes its volatile profile in response to the exposure to pheromones. Studies on the sawfly’s olfactory perception of pine volatiles will further enhance our understanding of their behavior.

## Data Availability

All data are included in the manuscript.

## Supplementary Materials

The following supporting information can be downloaded at: https://www.mdpi.com/article/10.3390/insects15060458/s1, Table S1. Comparison of pupation of *Diprion pini* larvae that fed upon either egg-free or egg-laden *Pinus sylvestris* trees exposed to different pre-treatments with volatiles. Pupation of larvae that fed on pine trees without egg deposition: untreated (U + L), hexane-treated trees (H + L), or pheromone-treated trees (P + L). Pupation of larvae that fed on trees with sawfly egg deposition (E): untreated (U + E + L), hexane-treated (H + E + L), or pheromone-treated (P + E + L) trees. Data: Mean (± SE) number of pupae per tree; each treatment consisted of 7–10 trees and a maximum of 5 larvae per tree. Statistical differences were determined using the Mann–Whitney *U* test.
