# Supplementary material for: Pine Response to Sawfly Pheromones: Effects on Sawfly’s Oviposition and Larval Growth
Source: Insects. 2024 Jun 19;15(6):458. doi: 10.3390/insects15060458 (PMC11203435; doi:10.3390/insects15060458)
Supplement: Supplementary file 1 [file insects-15-00458-s001.zip › insects-3028824-supplementary.pdf]

Pine Response to Sawfly Pheromones: Effects on Sawfly's Oviposition and Larval Growth

Asifur Rahman-Soad[1], Norbert Bittner[1,2] and Monika Hilker[1]*

[1]Applied Zoology/Animal Ecology, Institute of Biology, Freie Universität Berlin, Berlin, Germany
[2]Institute of Translational Genomics, Helmholtz Zentrum München Deutsches Forschungszentrum für Gesundheit und Umwelt, Neuherberg, Germany

Asifur Rahman-Soad: ORCID: https://orcid.org/0000-0001-7387-5391

Norbert Bittner: ORCID: https://orcid.org/0000-0002-8588-185X

Monika Hilker: ORCID: http://orcid.org/0000-0001-7519-7395

*Corresponding author:

Monika Hilker, Applied Zoology/Animal Ecology, Institute of Biology, Freie Universität Berlin, Berlin, Germany, Email: monika.hilker@fu-berlin.de

Table S1. Comparison of pupation *of Diprion pini* larvae that fed upon either egg-free or egg-laden *Pinus sylvestris* trees exposed to different pre-treatments with volatiles. Pupation of larvae that fed on pine trees without egg deposition: untreated (U+L), hexane-treated trees (H+L), or pheromone-treated trees (P+L). Pupation of larvae that fed on trees with sawfly egg deposition (E): untreated (U+E+L), hexane-treated (H+E+L), or pheromone-treated (P+E+L) trees. Data: Mean (± SE) number of pupae per tree; each treatment consisted of 7-10 trees and maximum 5 larvae per tree. Statistical differences were determined using the Mann Whitney *U* test.

	Treatments							
	**U+L**	**U+E+L**		**H+L**	**H+E+L**		**P+L**	**P+E+L**
**Mean**	4.00	3.27		3.80	3.54		3.70	3.00
**SE**	0.235	0.406		0.290	0.340		0.366	0.330
***p* value**	0.2111			0.6402			0.1015	
